# Computed tomography-guided simultaneous coil localization as a bridge to one-stage surgery for multiple lung nodules: a retrospective study

**DOI:** 10.1186/s13019-019-0870-6

**Published:** 2019-02-26

**Authors:** Yu-Fei Fu, Yong-Guang Gao, Miao Zhang, Tao Wang, Yi-Bing Shi, Ya-Yong Huang

**Affiliations:** 1 Department of Radiology, Xuzhou Central Hospital, 199 Jiefang Road, Xuzhou, Jiangsu China; 2 Department of Thoracic Surgery, Xuzhou Central Hospital, 199 Jiefang Road, Xuzhou, Jiangsu China

**Keywords:** Computed tomography, Coil localization, Lung nodule

## Abstract

**Background:**

Video-assisted thoracoscopic surgery (VATS) has been widely used for diagnostic wedge resection of lung nodules. When VATS is performed for multiple lung nodules, preoperative localization for each target nodule is required. In this study, we evaluated the clinical effectiveness of computed tomography (CT)-guided simultaneous coil localization in one-stage VATS wedge resection for multiple lung nodules.

**Methods:**

Between November 2015 to March 2018, 19 patients with multiple target nodules underwent CT-guided simultaneous coil localization and one-stage VATS resection at our center. Data on the technical success of simultaneous localization and wedge resection, complications, and pathological results were collected.

**Results:**

A total of 43 nodules were localized. The localization was successfully achieved in 42 of 43 nodules (97.7%). The technique of simultaneous localization was successfully achieved in 18 of 19 patients (94.7%). Fifteen patients underwent unilateral lung localization and four patients underwent bilateral lung localization. Three patients (15.8%) experienced asymptomatic pneumothorax after localization. All patients successfully underwent one-stage wedge resection for all target nodules. The mean duration of one-stage VATS procedure was 171.8 ± 84.0 min. The mean volume of blood loss was 94.2 ± 58.0 mL. Three patients experienced pleural effusion after VATS. During a follow-up of 6–31 months (median 18 months), no patient developed new lung nodules or distant metastasis.

**Conclusions:**

Preoperative simultaneous coil implantation is a safe and simple method for localization of multiple lung nodules. Simultaneous coil localization could effectively guide a one-stage VATS diagnostic wedge resection procedure.

## Background

Lung nodules are usually detected by chest computed tomography (CT). Among the lung nodules which were confirmed by pathologic examination, the mean rate of malignancy was approximately 70% [1–4]. Video-assisted thoracoscopic surgery (VATS) has been widely used for diagnostic wedge resection of lung nodules due to its minimal invasive feature [3–8]. Furthermore, the rate of wedge resection of lung nodules successfully increased when it was used along with preoperative localization technique [5].

Among the patients with lung nodules, some were also presented with multiple lung nodules. Except for the typical metastatic nodules, multiple primary lung cancers or precancerosis were also observed in patients with multiple lung nodules [3–8]. When VATS is performed for multiple lung nodules, simultaneous localization for each target nodule is required [7, 8]. Currently, not much information is available about the research on simultaneous multiple localization, although some previous studies included both single and multiple localizations [3–8].

In this study, we evaluated the clinical effectiveness of CT-guided simultaneous coil localization in one-stage VATS wedge resection for multiple lung nodules.

## Methods

This retrospective study was approved by our institutional review board, that also waived the requirement of informed consent for the use of the patients’ medical data.

### Patients

From November 2015 to March 2018, 19 patients with multiple target nodules underwent CT-guided simultaneous coil localization and one-stage VATS resection at our center (Table 1). Three of the 19 patients simultaneously had definitely diagnosed lung cancer (diagnosed by lung biopsy) in the lobe which was apart from the target nodules, one patient previously underwent resection of rectal cancer, and one patient who had previously undergone resection of hepatocarcinoma. The decision of resection of the lung nodules was made after discussing with the thoracic surgeons, oncologists, and radiologists.

**Table 1:** Baseline data of the 19 patients

	Values
Age (years)	56.3 ± 10.7 (27–76)
Gender (male/female)	10/9
Smoking history	2
Tumor history	2
Tumor marker	
Neuron-specific enolase (ng/L)	12.1 ± 2.0 (8.8–15.9)
Carcinoembryonic antigen (ug/ml)	1.7 ± 0.8 (0.3–3.6)
Squamous cell carcinoma antigen (ug/L)	1.1 ± 0.5 (0.5–2.3)
sCyfra21–1 (ng/ml)	1.9 ± 0.7 (1.0–3.4)

The inclusion criteria were: (a) multiple target lung nodules; (b) each lesion of diameter ≤ 3 cm; and (c) lesion-pleura distance ≤3 cm. The exclusion criteria were: (a) a lesion diameter < 3 mm; (b) typical benign lesion (e.g., calcification); (c) typical lung metastasis; and (d) extra-pulmonary metastasis.

### Coil localization

**Fig. 1:**
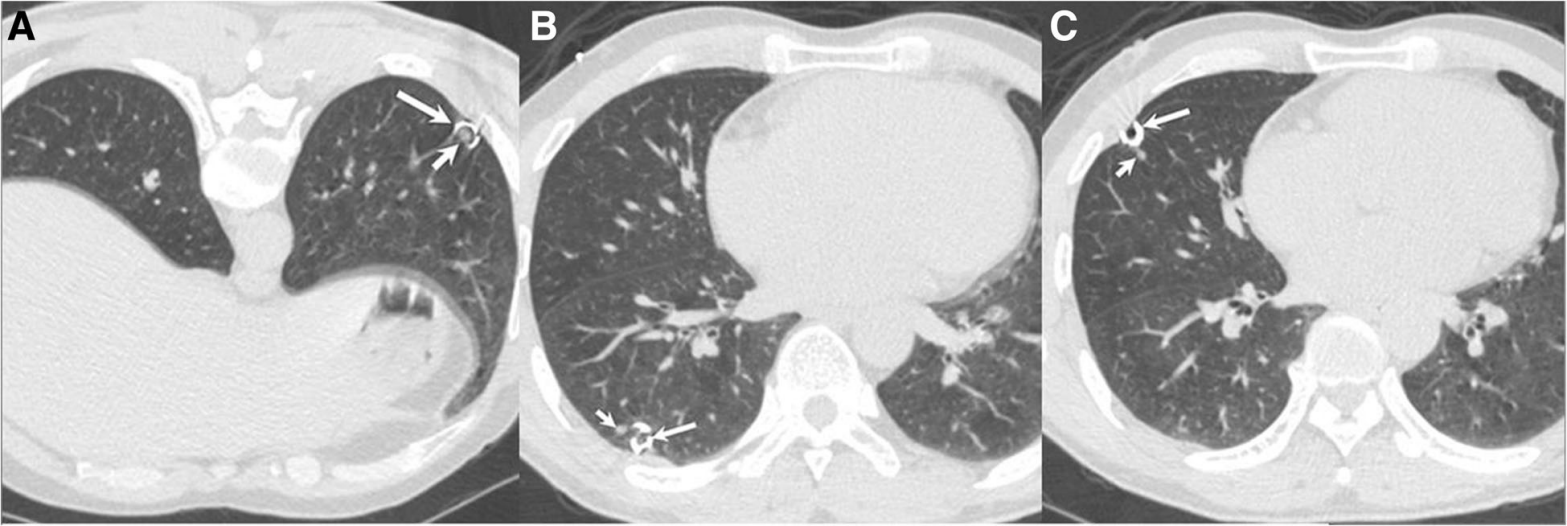
A 47-year-old female underwent simultaneous coil localization (long arrows) for three lung nodules (short arrows) in left lower (**a**), right lower (**b**), and right middle (**c**) lobes

All procedures were performed under the guidance of CT. The location in the patient was set according to the position of the nodule. Before localization, a preoperative chest CT scan was performed to confirm the position of the lesions. The puncture pathway was planned according to the position of the target lesions. After administration of local anesthesia with lidocaine, an 18 G coaxial needle (Precisa, Roma, Italy) was punctured into the lung and the tip of the needle was placed within 1 cm near the lesion. A coil of 50 mm length and 0.038- in. diameter (Cook, Bjaeverskov, Denmark) was partially inserted by “leaving-coil-end implantation” method [6] into the lung tissue via the needle sheath. The tail of the coil remained above the visceral pleura depending on the distance between the lesion and the pleura. Finally, the placement of the coil was confirmed through CT examination (Fig. 1) and its complications were further evaluated.

All nodules were simultaneously localized in a one-stage CT-guided procedure.

### Wedge resection

All patients underwent VATS within 24 h after coil localization. The wedge resection was performed based on visualization of the end tail of the coil above the visceral pleura. If the coil could not be seen during the VATS procedure, palpation was attempted to locate the coil. A long oval forcep was inserted from the incision and massive lung tissue containing the nodule and coil was lifted close to the incision. Then the coil was detected by the finger touch. If the coil could still not be palpated, the lobectomy was performed.

All patients underwent one-stage wedge resection of all target nodules. The cutting edge was at least 2 cm from the coil. The resected tissues were dissected to confirm the lesion, which were then sent for fast pathologic examination. No further resection was performed if the lesion were diagnosed as benign, precancerous, adenocarcinoma in situ (AIS), minimally invasive adenocarcinoma (MIA) or metastatic. However, lymph node sampling or systemic lymph node dissection was required for patients with MIA. Subsequently, if invasive carcinoma was detected, additional lobectomy with systematic lymph node dissection was performed.

If a patient was found with multiple invasive carcinomas in different lobes, additional lobectomy was performed for the lesion in the highest stage of cancer.

### Assessments

The coil localization was considered technically successful if the end tail of the coil was visible during the VATS procedure. A malignant or precancerous lesion was considered to be a positive pathologic result and a benign lesion was considered to be a negative pathologic result.

All patients underwent thoracic CT at 3, 6, 12 months and then yearly after surgery to detect if there were newly developed lung nodules.

### Statistical analysis

Statistical analysis was performed by SPSS version 16.0 (SPSS, Chicago, IL, USA). Quantitative variables were demonstrated as the mean ± standard deviation (SD) or median, and categorical variables were mentioned in the terms of frequencies or percentages.

## Results

### Localization procedure

A total of 50 undiagnosed lung nodules were found in 19 patients, 43 of which were localized (Table 2). Fourteen patients were localized with all lung nodules and five patients were localized with part of the nodules. Among the remaining seven nodules in the five patients, six nodules were less than 3 mm and one nodule decreased in size after a follow-up of 3 months. All patients underwent simultaneous coil localization for all target nodules. Technically, the localization was successful in 42 of 43 nodules (97.7%) and simultaneous localization was successful in 18 of 19 patients (94.7%). One nodule could not be successfully localized because the coil was not visible during the VATS procedure. One nodule was localized with two coils because the first coil was suspected to be completely inserted into the lung tissue.

**Table 2:** Characteristics of the nodules and localization

	Values
Nodule number	43
Patients with 2 nodules	15
Patients with 3 nodules	3
Patients with 4 nodules	1
Locations	
Right upper lobe	11
Right middle lobe	6
Right lower lobe	10
Left upper lobe	7
Left lower lobe	9
Diameter (mm)	7.4 ± 4.6 (3–25)
Nodule-pleura distance (mm)	5.0 ± 5.6 (0–26)
Localization	
Unilateral lung localization	15
Bilateral lung localization	4

Fifteen patients underwent unilateral lung localization and four patients underwent bilateral lung localization.

The mean duration of each localization procedure was 14.2 ± 5.1 min. Three patients (15.8%) experienced asymptomatic pneumothorax after localization. The pneumothorax did not influence the VATS procedure.

### One-stage wedge resection

All patients successfully underwent one-stage wedge resection of all target nodules (Table 3). Although one nodule was invisible during the VATS procedure, the coil was successfully localized through palpation. No patient was advised thoracotomy during the surgery. A negative surgical margin was confirmed for all wedge tissues.

**Table 3:** Details of the VATS procedure of the 19 patients

No.	Nodules location	Nature	Surgical type	Duration of one-stage VATS (min)	Blood loss (ml)	Pathological results
1	RU/RL/RL	GGO/Solid/Solid	Wedge/Wedge/Wedge	170	100	Benign/Benign/Benign
2	LL/RM/RL	GGO/mGGO/mGGO	Wedge/Wedge/Wedge	180	100	Benign/Benign/Benign
3	RU /RM	Solid/Solid	Wedge/Wedge	205	100	PC/PC
4	RU /RL	GGO/GGO	Wedge/Wedge	60	50	AIS/PC
5	RU /LU	GGO/GGO	Wedge/Wedge	90	50	PC/PC
6	RU /RL	mGGO/mGGO	Wedge+ lobectomy/Wedge	230	50	IA/MIA
7	LL/RU	Solid/GGO	Wedge/Wedge	130	100	Benign/PC
8	RM/RL	GGO/GGO	Wedge/Wedge	70	50	PC/PC
9	LL/ LL	GGO/GGO	Wedge/Wedge^a^	360	200	AIS/AIS
10	RL/RU	Solid /mGGO	Wedge/Wedge	145	100	Benign/PC
11	LL/RM	Solid/Solid	Wedge/Wedge	200	200	Benign/Benign
12	RL/RM	GGO/Solid	Wedge/Wedge^a^	195	100	Benign/MIA
13	RU/RU/RU	GGO/GGO/GGO	Wedge/Wedge/Wedge^a^	310	200	Benign/PC/MIA
14	LU/ LL	GGO/GGO	Wedge/Wedge	80	10	AIS/PC
15	LU/ LU	GGO/Solid	Wedge/Wedge	100	50	AIS/Metastasis
16	LL/LU	Solid/Solid	Wedge/Wedge^b^	165	150	Benign/PC
17	LU/ LL	GGO/Solid	Wedge/Wedge	80	60	AIS/IA
18	LU/ LL	Solid/Solid	Wedge/Wedge	270	20	Benign/Benign
19	RL/RL/RM/RU	GGO/GGO/GGO/mGGO	Wedge/Wedge/Wedge/Wedge	225	100	AIS/AIS/Benign/AIS

*VATS* video-assisted thoracoscopic surgery, *RU* right upper, *RM* right middle, *RL* right lower, *LU* left upper, *LL* left lower, *GGO* ground-glass opacity, *mGGO* mixed GGO, *PC* precancerosis, *AIS* adenocarcinoma in situ, *MIA* minimally invasive adenocarcinoma, *IA* invasive adenocarcinoma. ^a^: These patients had synchronous definite diagnosed lung cancer and they underwent lobectomy and wedge resection during a single-stage VATS procedure. ^b^: This wedge resection was performed based on the palpation of the coil

The fast-pathologic results of the 43 nodules included invasive adenocarcinoma (*n* = 2), MIA (*n* = 3), AIS (*n* = 9), metastasis (*n* = 1), precancerous (*n* = 12), and benign (*n* = 16). No patient had synchronous invasive adenocarcinomas. One patient underwent subsequent right upper lobectomy due to invasive adenocarcinoma, and another elderly patient declined lobectomy due ageing.

The three patients who were detected with synchronous definite diagnosed lung cancer, all underwent lobectomy and wedge resection during a single-stage VATS procedure.

The mean duration of one-stage VATS procedure was 171.8 ± 84.0 min. The mean volume of blood loss was 94.2 ± 58.0 mL. Three patients experienced pleural effusion after VATS.

During the follow-up of 6–31 months (median 18 months), no patient developed new lung nodules or distant metastasis. Among the seven residual nodules in four patients, five nodules were stable and two nodules disappeared.

## Discussion

This study evaluated the feasibility of CT-guided simultaneous coil localization of multiple lung nodules and the clinical effectiveness of coil localization in one-stage VATS wedge resection procedure. Technically, the success rates of simultaneous localization and one-stage VATS wedge resection were 94.7 and 100%, respectively. These results demonstrate that: (a) CT-guided simultaneous coils implantation is a simple and effective method for localization of multiple lung nodules; (b) simultaneous coil localization can effectively guide a one-stage VATS wedge resection procedure.

In lung cancer cases, synchronous multiple lung cancers have been reported in some studies with an incidence rate of 1–8% [9]. Patients with early-stage multiple lung cancers can benefit from multiple VATS resection [10–13]. Theoretically, simultaneous resection is better compared with a two-stage procedure in terms of reducing the risk of disease progression [13].

Accurate diagnosis is critical in the management of lung nodules and a precise pathologic diagnosis is not possible only through image analysis. Although CT-guided cutting needle biopsy can provide a high diagnostic accuracy for lung nodules, it still had a false-negative rate of 5.6–9.6% [14, 15]. Moreover, some small lung nodules may be missed because of technical limitations [16]. In this study, the mean diameter of resected nodules was 7.4 mm, and it was very difficult to perform a biopsy. The diagnostic wedge resection is the gold standard for diagnosis of lung nodules. Furthermore, wedge resection is also considered as a curative treatment for precancerosis, and AIS [3–8]. Some researchers are also of the view that wedge resection could be used for the curative treatment of MIA [3]. In the past, the wedge resection was performed based on the palpation of the lung nodule [5], which was unable to detect some small or sub-solid nodules due to their small size and soft nature. When the wedge resection cannot be performed, the lobectomy should be performed [4], although, unnecessary lobectomy should be avoided to preserve the respiratory function.

For patients with multiple lung nodules, multiple localization can significantly benefit the wedge resection of each nodule [7, 8]. Various methods and materials have been used for preoperative localization including methylene blue, hook-wire, radio-label and coil [3–8]. Tseng et al. [7] and Iguchi et al. [8] used methylene blue and hook-wire, respectively, to localize the multiple lung nodules, with the technical success rates of 99 and 96%, respectively. Correspondingly, the technical success rate in our study was 94.7% and comparable to that of these two studies. However, the earlier methods have some notable disadvantages. Localization of methylene blue is difficult because of its rapid diffusivity [7]. Hook-wire is usually limited by a high incidence of wire dislodgement that may cause pneumothorax, hemorrhage, and chest pain [8]. Radio-label localization guided VATS requires intraoperative fluoroscopy, which brings patients to radiation exposure [17].

Coil localization has been reported in several studies [4–6]. In our study, the coil was inserted by “leaving-coil-end implantation” technique. The end tail of the coil can be easily detected during the VATS. However, this technique requires a well-developed skill and extensive experience. In this study, coil localization failed in one nodule (1/43, 2.3%), but this nodule was also successfully removed by wedge resection based on the successful palpation of the coil. Su et al. [6] also reported 51 cases of entire implantation of the coil and the results demonstrated successful VATS wedge resection in all cases. Furthermore, the coil localization can also help a pathologist to find the lesions in the resected tissue.

In this study, the incidence rate of pneumothorax was 15.8%. This rate was lower than that mentioned in a previous study (89.5%) about hook-wire localization for multiple lung nodules and comparable to that mentioned in a previous study (21.6%) about coil localization for multiple lung nodules [8, 18]. In addition, Li et al. [18] also found no significant difference in pneumothorax (21.6% vs 14.1%, *P* = 0.179) between multiple and single coil localization groups. Kadeer et al. [19] used a modified hook-wire implantation technique which comprised a row of metal wires, perpendicular insertion, simultaneous release of hook-wire, and a lateral position to localize multiple lung nodules. Compared to the conventional hook-wire insertion, the modified technique can significantly decrease the incident rate of pneumothorax but cannot decrease the incident rate of hemorrhage [19]. Iguchi et al. [8] considered that bilateral hook wire placements should not be performed during one session because bilateral pneumothoraxes may lead to a lethal outcome. However, in this present study, we successfully performed one-stage bilateral coil localization for four patients and no major complication occurred. Thus, we may surmise that one-stage bilateral coil localization is a safe procedure.

Recently, some researchers performed the bronchoscopy-guided dye marking for VATS of lung nodules [20, 21]. This technique may avoid the CT-guided percutaneous transthoracic procedures related complications. However, this technique usually requires real-time fluoroscopic guidance, which can increase the radiation exposure [20].

Based on the pathologic diagnoses from the wedge resection, one patient underwent resection of one lobe due to the invasive adenocarcinoma. In addition, three patients directly underwent VATS lobectomy due to the confirmed diagnosis of lung cancer. The residual nodules were radically resected through wedge resection. This treatment strategy preserved the maximum respiratory function. In one patient, one nodule was diagnosed as invasive adenocarcinoma, but this patient only underwent wedge resection due to the older age. However, during the follow-up, this patient did not develop new lung nodules or distant metastasis.

This study gave encouraging results, although it has some limitations. The first and a major limitation of this study is its retrospective nature, thus, the selected bias definitely existed. Second, there was no control group in this study. Therefore, we could not compare this method to other preoperative localization methods for multiple lung nodules. Third, the period of follow-up was not long. Although no patient developed new lung nodules or distant metastasis, further follow-up results are definitely required.

## Conclusion

In conclusion, although further prospective, randomized controlled trials are needed, the results of this study indicate that preoperative simultaneous coil implantation is a safe and simple method for localization of multiple lung nodules and can effectively guide a one-stage VATS diagnostic wedge resection procedure.

## Abbreviations

AIS: Adenocarcinoma in situ
CT: Computed tomography
GGO: Ground-glass opacity
MIA: Minimally invasive adenocarcinoma
VATS: Video-assisted thoracoscopic surgery
